# Cardiac evaluation of patients with juvenile dermatomyositis

**DOI:** 10.1038/s41390-024-03336-8

**Published:** 2024-06-22

**Authors:** Gökmen Akgün, Betül Sözeri, Eviç Zeynep Başar, Nihal Şahin, Yunus Emre Bayrak, Kadir Ulu, Hüseyin Salih Güngör, Mustafa Doğan, Taliha Öner, Mehmet Karacan, Kadir Babaoğlu, Yonca Anık, Hafize Emine Sönmez

**Affiliations:** 1 Department of Pediatric Cardiology, City Hospital, Kocaeli, Turkey; 2 Department of Pediatric Rheumatology, University of Health Sciences, Ümraniye Research and Training Hospital, Istanbul, Turkey; 3 Department of Pediatric Cardiology, Kocaeli University, Faculty of Medicine, Kocaeli, Turkey; 4 Department of Pediatric Rheumatology, Kocaeli University, Faculty of Medicine, Kocaeli, Turkey; 5 Department of Pediatric Cardiology, University of Health Sciences, Ümraniye Research and Training Hospital, Istanbul, Turkey; 6 Department of Radiology, Kocaeli University, Faculty of Medicine, Kocaeli, Turkey

## Abstract

**Background:**

The present study aims to evaluate possible cardiac involvement in juvenile dermatomyositis (JDM) patients by conventional methods and cardiac magnetic resonance imaging (MRI) along with a systematic review of the literature on cardiac features in JDM.

**Methods:**

The study group consisted of JDM patients who underwent cardiac MRI. We conducted a systematic review of the published literature involving JDM patients with cardiac involvement.

**Results:**

In the present study, although baseline cardiologic evaluations including electrocardiography and echocardiography were within normal limits, we showed late gadolinium enhancement on cardiac MRI in 3 of 11 JDM patients. In the literature review, we identified 25 articles related to cardiac involvement in JDM. However, none of them, except one case report, included cardiac MRI of JDM patients.

**Conclusion:**

Cardiac abnormalities have been reported among the less frequent findings in patients with JDM. Cardiovascular complications during the long-term disease course are a leading cause of morbidity and mortality in these patients. Early detection of cardiac involvement by cardiac MRI in patients with JDM and aggressive treatment of them may improve the clinical course of these patients.

**Impact:**

- The myocardium in patients with JDM may be involved by inflammation.
- Myocardial involvement may be evaluated by using contrast-enhanced cardiac MRI.
- This is the first study evaluating cardiac involvement by cardiac MRI in JDM patients.
- MRI may show early cardiac involvement in patients whose baseline cardiologic evaluations are within normal limits.
- Early detection of cardiac involvement by cardiac MRI may improve the long-term prognosis of patients with JDM.

## Introduction

Juvenile dermatomyositis (JDM) is the most common idiopathic inflammatory myopathy presenting with the characteristic rash and proximal muscle weakness during childhood.^1^ Although proximal muscle weakness and skin findings are the predominant features of the disease, cardiac findings may be accompanied at initiation or during the course of the disease.^2^ Cardiac involvement in JDM is usually underestimated and studies regarding this issue are scarce. However, cardiac involvement may cause morbidity and mortality in the long-term period. Nonspecific sinus tachycardia is the most frequent cardiac abnormality in JDM. Left ventricular diastolic and systolic dysfunction, hypertension, atherosclerosis, coronary artery disease, and metabolic syndrome may also occur.^2^ Increasingly, magnetic resonance imaging (MRI) studies begin to take an important place both in the diagnosis of the disease and detection of subclinical organ involvement. Adult studies showed that cardiac MRI may help to detect subclinical myocardial involvement.^3,4^ However, there is no data about cardiac MRI findings in JDM patients. The present study aims to evaluate possible cardiac involvement in JDM patients by conventional methods and cardiac MRI along with a systematic review of the literature on cardiac features in JDM.

## Material and methods

This cross-sectional study was conducted between June 2022 and December 2022. Patients who were followed up with a diagnosis of JDM were enrolled in the study. All patients fulfilled the Bohan and Peter^5,6^ or EULAR/ACR 2017 classification criteria.^7^ Demographic data, clinical manifestations, laboratory findings, treatments, and outcomes were obtained from patient charts. The disease activity was evaluated with the Childhood Myositis Assessment Scale (CMAS).^8^

Initially, a standard 12-lead electrocardiogram (ECG) was performed in all patients. Subsequently, all of them were examined using transthoracic echocardiography (GE Vivid E9 ultrasound system, General Electric Healthcare, Horten, Norway). B-Mode and M-Mode echocardiographic images were obtained. Finally, all patients underwent cardiac MRI in a 1.5 T MR scanner (Philips Gyroscan Intera Master; Philips, Eindhoven, The Netherlands) equipped with a 30 mT/m maximum gradient strength and 150 mT/m/ms slew rate. Data were collected by utilizing a synergy body coin with the patient in the supine position. The vital signs of patients were monitored and recorded throughout the MR examination. The left ventricle (LV) was evaluated in the standard 17-segment model^9^ (Supplementary Fig. 1). All images were analyzed by an experienced pediatric cardiologist (KB) and radiologist (YA).

The study respected the guidelines of the Helsinki Declaration concerning medical research in humans and received local Ethics Committee approval. Informed consent was obtained from each patient and/or parents.

### Systematic review of the literature

A systematic review of the literature on cardiac involvement in JDM was conducted. Relevant documents were searched according to Preferred Reporting Items for Systematic Reviews and Meta-Analyses (PRISMA) principles by using Medline and PubMed databases (Supplementary Fig. 2).^10^ The following keywords were used: (“Juvenile dermatomyositis” OR “JDM” OR “Dermatomyositis, Juvenile” OR “Juvenile Myositis” OR “Myositis, Juvenile”) AND (“cardiac involvement” OR “cardiac manifestation” OR “heart involvement” OR “heart manifestation”). Articles only in English were included in the search. All related articles, including randomized and nonrandomized controlled trials, observational studies (case-control, cohort studies, and case series), and single case reports, were included. Two authors (GA and HES) independently reviewed all possibly eligible studies. Finally, discrepancies were resolved by discussion with the two experienced authors (KB and BS).

## Results

### Baseline characteristics of our patients

A total of 11 patients were included in the study. Four (36.4%) were male and 7 (63.6%) were female. The median age at evaluation was 11 (7–17) years. The median duration of the disease was 24 (1–102) months. All the patients had heliotrope rash and proximal muscle weakness. Gottron papules were present in 10 patients, calcinosis in 2, and lipodystrophy in 2, concomitantly. The median CMAS score was 35 (22–48) at the time of diagnosis and 52 (41–52) at the time of cardiac evaluation. None of the patients had pulmonary or gastrointestinal involvement. Anti-nuclear antibody (ANA) was positive in 10 patients, anti-transcription intermediary factor 1γ (Anti-TIF1γ) in 2, anti-NXP2 in 1, and anti-Mi2 alfa and beta in 1 patient. Nailfold capillaroscopy findings revealed scleroderma pattern in 5 patients and non-scleroderma pattern in 5 patients (Table 1). None of them had additional cardiac risk factors including smoking, obesity, hypertension, dyslipidemia, and metabolic syndrome.

**Table 1:** Demographical, clinical, and cardiac findings of patients.

	Patients without myocardial LGE (*n* = 8)	Patients with myocardial LGE (*n* = 3)
Age, year	13 (7–17)	7.5 (7–15)
Sex (Female/Male)	5/3	3/0
Duration of the disease, months	48 (9–102)	3 (3–12)
Heliotrope rash, *n* (%)	8 (100)	3 (100)
Gottron papules, *n* (%)	8 (100)	3 (100)
Calcinosis, *n* (%)	0 (0)	0 (0)
Lipodystrophy, *n* (%)	1 (12.5)	0 (0)
Pulmonary involvement, *n* (%)	0 (0)	0 (0)
Gastrointestinal Involvement, *n* (%)	0 (0)	0 (0)
Echocardiography abnormality, *n* (%)	0 (0)	0 (0)
Electrocardiography abnormality, *n* (%)	0 (0)	0 (0)
CMAS (at diagnosis)	34 (22–48)	35 (30–40)
CMAS (at the time of cardiac involvement)	52 (52–52)	52 (52–52)
Myositis-specific or associated antibody	Anti-TIF 1 = 2 Anti- NXP2 = 1	Anti Mi-2-α ve β = 1
ANA, *n* (%)	7 (87.5)	3 (100)
Capillaroscopy abnormality, *n*	Scleroderma pattern = 4 Non-scleroderma pattern = 4	Scleroderma pattern = 1 Non-scleroderma pattern = 1
Drugs (previously used), *n*	Steroid = 8 Methotrexate = 8 IVIG = 4 Cyclophosphamide = 1 Hydroxychloroquine = 7 Tofacitinib = 1 Tocilizumab = 2 Rituximab = 1 MMF = 3	Steroid = 3 Methotrexate = 3 Hydroxychloroquine = 3
Drugs (at the time of evaluation), *n*	Methotrexate = 4 Cyclosporine = 1 MMF = 2 Tocilizumab = 1 Hydroxychloroquine = 6	Methotrexate = 3 Hydroxychloroquine = 3

*ANA* anti-nuclear antibody, *CMAS* childhood myositis assessment scale, *IVIG* intravenous immunoglobulin, *LGE* late gadolinium enhancement, *MMF* Mycophenolate mofetil, *MRI* magnetic resonance imaging.

### Cardiac features of our patients

**Fig. 1:**
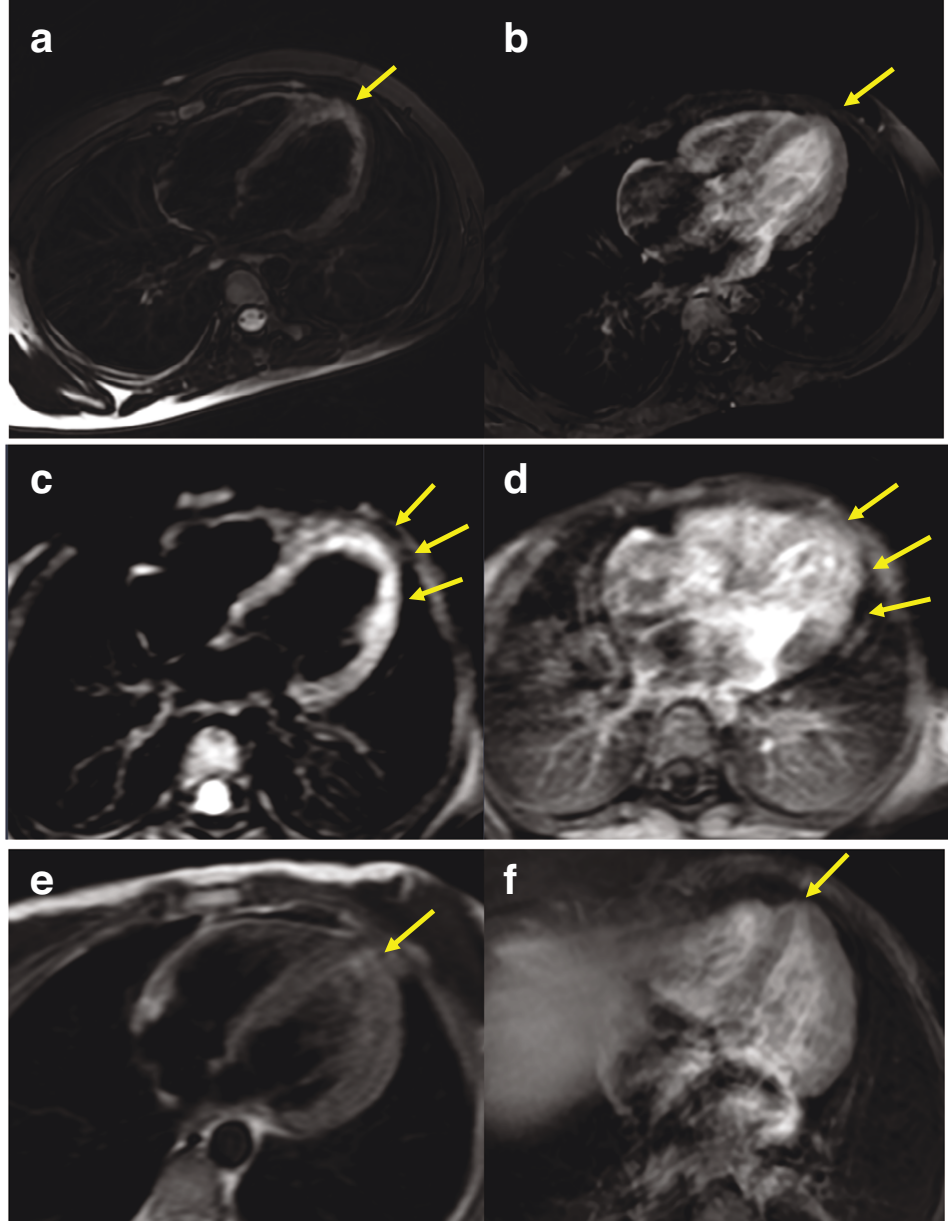
Cardiac magnetic resonance images of patients. Myocardial images of T2 scans (**a**, **c**, **e**) and late gadolinium enhancement T1 scans (**b**, **d**, **f**) on cardiac MRI. Cardiac MRI axial plane: (**a**, **c**, **e**) are T2 weighted fat saturated images and (**b**, **d**, **f**) are postcontrast late enhancement T1 weighted images. Myocardial edema is seen on segment 17 (apex) on images (**a**) and (**e**) and on segments 1, 2, 5, 6, 9, 10, 11, and 14 (basal anterior, basal anteroseptal, basal inferolateral, basal anterolateral, mid inferoseptal, mid inferior, mid inferolateral and apical septal) on the image (**c**). On postcontrast images (**b**) (**d**) and (**f**) contrast enhancement is seen on the segments parallel with myocardial edema areas on T2W-fat-saturated images.

Electrocardiographic examination showed normal sinus rhythm with normal QRS voltage and axis in all patients. None of them had arrhythmia at the time of evaluation. Conventional echocardiographic parameters were within the normal range (Table 1). Three of 11 patients (27.2%) had myocardial late gadolinium enhancement (LGE) on cardiac MRI similar to the appearance in myocarditis. Segment 17 (apex) was involved in 2 patients, and segments 1,2 5,6,9,10,11,14 (basal anterior, basal anteroseptal, basal inferolateral, basal anterolateral, mid inferoseptal, mid inferior, mid inferolateral, and apical septal) were involved in 1 patient (Fig. 1). Furthermore, one patient showed impaired right ventricular ejection fraction (EF) which was not shown in conventional echocardiography. Patients with myocardial late gadolinium enhancement were younger than others, but there were no differences between these two groups in terms of clinical and laboratory findings.

### Cardiac features of patients with JDM in the literature

Supplementary Fig. 2 lists the schematic analyzes of the systematic literature review. At first, 84 related articles were identified. After the title and abstract review, 25 articles were included.^11–35^ Of these 25 articles, 7 were case report and 18 were observational studies (Table 2). However, none of them, except one, included cardiac MR findings of JDM patients. Stewart et al.^32^ reported a 14-year-old girl diagnosed with JDM who had first-degree heart block and low voltages on ECG. Her disease evolved into multisystem involvement during hospitalization and treatment. Conduction disturbance progressed to atrial fibrillation. Her echocardiographic examination and cardiac MRI were normal. The disease course of the patient was complicated by macrophage activation syndrome. She was treated successfully with immunosuppressive therapy. Karaca et al.^16^ reported another case report of conduction disturbance in an 11-year-old boy with JDM. The patient had sinus bradycardia with a heart rate of 40–44 per minute. After treatment with intravenous immunoglobulin (IVIG) and steroids, bradycardia was resolved spontaneously. A case-control study by ref. ^13^ including 25 patients with JDM reported one patient whose initial presentation was cardiac failure due to myocarditis and another two patients with asymptomatic ECG abnormalities. Ghosh et al.^30^ reported a 10-year-old girl with a complete heart block secondary to JDM. She had normal systolic and diastolic functions. After treatment with methylprednisolone, ECG returned to sinus rhythm. Another case-control study by ref. ^19^ reported that diastolic dysfunction detected by tissue Doppler imaging was present in 22% of patients with JDM, pericarditis in 11%, and ECG abnormalities in 17% during 16 years of clinical follow-up. In their ensuing study, ref. ^21^ reported that patients with JDM had systolic dysfunction represented by decreased long-axis strain on color tissue Doppler echocardiography. Barth et al.^23^ investigated 55 JDM patients aged 6–48 years using 24-h ambulatory Holter ECG and stated that patients with JDM had decreased heart rate variability (HRV). HRV is related to the heart rate. Insufficient response of inflamed myocardium and conduction tissue to vagal and sympathetic stimulus may be the reason for decreased HRV. Cantez et al.^27^ reviewed retrospectively 105 patients with JDM. They found that ECG abnormalities (prolonged QTc and PR interval, bundle branch block) were present in 6% of the patients and echocardiographic abnormalities (atrioventricular-semilunar valve regurgitations) in 25%. However, their ECG and echocardiographic findings were mild or trivial and unlikely to be linked to the underlying disease. Diniz et al.^31^ investigated 35 patients with JDM using conventional 2D and speckle-tracking echocardiography. They found that patients with JDM had lower LV global peak longitudinal systolic strain as well as lower peak circumferential systolic strain values than controls, although all the patients had normal LV ejection fraction (EF) on 2D conventional echocardiography. These results indicate that speckle-tracking echocardiography may be more sensitive than conventional 2D echocardiography in detecting early subclinical myocardial impairment in JDM patients. Diastolic dysfunction represented by low early diastolic tissue velocity (e’) and systolic dysfunction represented by decreased longitudinal strain were reported by ref. ^34^ including 57 patients with JDM. Patients with JDM generally have subclinical silent cardiac involvement during childhood, although there are few case reports of pericardial tamponade resulting in congestive heart failure which was treated by open drainage^11^ and ventricular arrhythmias treated by amiodarone.^36^ Overt cardiovascular disease and death resulting from cardiovascular involvement in JDM are more common in adult populations during long-term follow-up.

**Table 2:** Summary of studies focusing on cardiac involvement in juvenile dermatomyositis.

First author, year of publication	Type of article	Number of patients	Sex	Age at diagnosis	Age at cardiac involvement	Type of cardiac involvement	Treatments	Follow-up/ Outcome
The present study	Observational study	11	8 female/ 3male	11 (7–17) years	13 (7–17) years	Intramyocardial LGE in 3 patients	Prednisone, hydroxychloroquine, IVIG, methotrexate	Resolved in all patients on 6th- month cardiac MRI
Pereira et al.^11^	Case report	1	Female	6-year-old	8-year-old	Pericardial Tamponade	Azathioprine, prednisone, chloroquine diphosphate	2 years Inactive disease
Jimenez et al.^12^	Case report	1	Female	6-year-old	6-year-old	Left ventricular dilation and transmural hemorrhagic necrosis	NA	3.5 day/ Exitus
Shehata et al.^13^	Observational study	3 of 25 had cardiac involvement	NA	NA	NA	Myocarditis with cardiac failure in 1 patient, conduction defect in 2 patients	NA	NA
Hicks et al.^14^	Observational study	14	11 female/ 3 male	NA	11.2 ± 3.0 years	Reduced peak heart rate and aerobic capacity	Prednisone, hydroxychloroquine, IVIG, methotrexate, cyclosporine	NA/NA
Takken et al.^15^	Observational study	15 patients	10 female/ 5 male	NA	9.56 ± 2.7	Impaired aerobic exercise capacity	Prednisone, Methotrexate, Cyclophosphamide	NA/NA
Karaca et al.^16^	Case report	1	Male	11-year-old	11-year-old	Sinus bradycardia	Intravenous Immunoglobulin, pulse methylprednisolone, methotrexate	NA/ bradycardia Resolved
Sallum et al.^17^	Observational study	8 of 54 had cardiac involvement	NA	NA	NA	NA	NA	NA/ Cardiac involvement was associated with increased frequency of calcinosis
Sakurai et al.^18^	Case report	1	Male	9-year-old	9-year-old	Cardiac failure, rapidly Progressive Interstitial Lung Disease	Methylprednisolone, cyclophosphamide, cyclosporin A	3 months/ Exitus
Schwartz et al.^19^	Observational study	59 patients	36 Female/23 Male	8.9 (2.1–19.2) years	21.5 (6.7–55.4) years	Diastolic dysfunction (E/E’ elevated in patients with JDM compared to controls)	NA	16.8 (2.0–38.1) years/NA
Rider et al.^20^	Observational study	374 patients	NA	NA	NA	34 (9.1%) chest pain 56 (15%) abnormal ECG or ECHO findings 36 (9.6%) palpitation	NA	NA/Cardiac involvement was associated with the presence of anti-SRP autoantibodies
Schwartz et al.^21^	Observational study	59 patients	36 Female/23 Male	8.9 (2.1–19.2) years	21.5 (6.7–55.4)	Impaired cardiac systolic function	NA	16.8 (2.0–38.1) years/NA
Schwartz et al.^22^	Observational study	59 patients	36 Female/23 Male	8.9 (2.1–19.2) years	21.5 (6.7–55.4)	Eotaxin and MCP-1 were associated with cardiac dysfunction	NA	16.8 (2.0–38.1) years/NA
Barth et al.^23^	Observational study	55 patients	34 Female /21 Male	NA	19.8 (6.7–48.9) years	Increased HR and decreased HRV	NA	13.5 (range 2–34) years/NA
Khera et al.^24^	Case report	1	Male	7-year-old	7-year-old	Bradycardia and prolongation of PR interval	Methylprednisolone	15th day/ bradycardia Resolved
Saini et al.^25^	Observational study	2 of 39 had cardiac involvement	NA	NA	NA	NA	NA	NA/Cardiac involvement was associated with calcinosis
Berntsen et al.^26^	Observational study	59 patients	36 female/ 23 male	NA	25.2 (12.5) years	Reduced submaximal exercise capacity	NA	NA
Cantez et al.^27^	Observational study	6 of 69 patients had cardiac involvement	NA	6.9 (2.0–17.6) years	6.9 (2.0–17.6) years	Prolonged QTc in 5, Prolonged PR + wide QRS in 1	NA	10.2 years (7 months – 15.8 years)/NA
Silverberg et al.^28^	Observational study	NA	NA	NA	NA	Increased risk of cardiovascular comorbidities	NA	NA
Barth et al.^29^	Observational study	58 patients	NA	NA	NA	10 (17%) pathologic ECG findings	NA	NA/ No associations between cardiac involvement and nailfold capillary density
Ghosh et al.^30^	Case report	1	Female	10-year-old	10-year-old	Complete heart block	Methylprednisolone	10^t^h day/normal sinus ritm
Diniz et al.^31^	Observational study	35 patients	11 female/ 24 male	6.2 (2.1–13.6) years	12.6 ± 0.7 years	Impaired longitudinal and circumferential strain in asymptomatic patients	Hydroxychloroquine, prednisone, azathioprine, methotrexate, mycophenolate mofetil, cyclosporine	NA/
Stewart et al.^32^	Case report	1	Female	14-year-old	14-year-old	First-degree heart block	Methylprednisolone, mycophenolate mofetil, IVIG, anakinra, cyclosporine, etoposide, tofacitinib	NA/Resolved
Witczak et al.^33^	Observational study	10 of 57 patients had cardiac involvement	5 female/5 male	NA	NA	NA	NA	No increased risk of developing concomitant cardiac/pulmonary involvement in patients
Witczak et al.^34^	Observational study	59 patients	36 female/ 23 male	NA	21.5 (15.4–34.8) years	Central body fat distribution was associated with impaired cardiometabolic measures	NA	21.6 (15.1–34.8) years/NA
Marstein et al.^35^	Observational study	39	18 female/ 11 male	NA	31.7 (10.3)	Increased risk of cardio-metabolic syndrome	NA	22.7 ± 8.9 years

*ECG* electrocardiogram, *ECHO* echocardiography, *HR* heart rate, *HRV* heart rate variability, *LGE* late gadolinium enhancement, *MRI* magnetic resonance imaging, *NA* not available

## Discussion

The myocardium in patients with JDM can be involved by inflammation just as the skeletal muscle. In the present study, we showed myocardial involvement by using contrast-enhanced cardiac MRI in 3 of 11 JDM patients whose baseline cardiologic evaluations including ECG and echocardiography were within normal limits. To our best knowledge, this is the first study evaluating cardiac involvement by cardiac MRI in JDM patients. Adults, with dermatomyositis and polymyositis frequently have cardiac involvement such as conduction abnormalities, atrial and ventricular arrhythmias, pericarditis, and myocarditis.^16^ Cardiac involvement in JDM may either develop acutely or manifest during the disease course. Systemic vasculopathy and elevated interferon signature play a central role in the pathogenesis of idiopathic inflammatory myopathies. Both factors result in endothelial dysfunction which leads to cardiovascular manifestation during the disease.^2^ Rhythm and conduction problems and myocarditis are the most observed cardiac abnormalities in the acute phase of the disease. Rider et al.^20^ evaluated 374 patients with juvenile idiopathic inflammatory myopathy. Abnormal ECG findings were present in 56 (15%) of the patients. However, the study did not specify the abnormal ECG findings. Cantez et al.^27^ showed prolonged QTc or prolonged PR in 6 of 69 JDM patients. Barth et al.^29^ demonstrated pathologic ECG findings in 10 (17%) of 58 JDM patients. Furthermore, patients presenting with varying degrees of heart block have been reported.^30,32^ Besides these acute complications, reduced aerobic capacity, systolic and diastolic dysfunction, and impaired cardiometabolic measures have been demonstrated in long-term follow-up.^14,15,19,21,26,28,33,35^

Current recommendation guidelines do not provide a recommendation on how often and by which method patients with JDM should be screened for cardiac aspects. Cardiac MRI may be a valuable prospective technique to detect subclinical myocardial involvement. However, it is recommended that cardiac MRI should only be performed in patients with abnormalities on ECG or echocardiography.^37^ Cardiac MRI studies conducted in recent years have revealed that myocardial involvement is higher than previously thought. For instance, Rosenbohm et al.^38^ demonstrated signs of myocardial inflammation in 62.3% of 53 polymyositis (PM)/dermatomyositis (DM) patients by cardiac MRI. In the aforementioned study, they reported that the lateral segments of the left ventricle were significantly more often affected than the anterior or septal segments. Another study by ref. ^4^ showed late gadolinium enhancement (LGE) involving around 5% of the mass of the myocardium in 9 of 16 adult patients with PM/DM. The most affected part of the heart was interventricular septum (*n* = 8) followed by lateral wall (*n* = 5), inferior wall (*n* = 3), apex (*n* = 2), and anterior wall of the left ventricle (*n* = 1). They also found that LGE was more common in patients with PM compared to those with DM.^4^ Diederichsen et al.^39^ evaluated cardiac abnormalities by cardiac MRI in newly diagnosed, untreated patients with idiopathic inflammatory myopathies. They detected systolic dysfunction by cardiac MRI in 2 of 14 patients with idiopathic inflammatory myopathies. Most recently, ref. ^40^ suggested that characteristics of cardiac dysfunction may be different between subgroups of idiopathic inflammatory myopathies, and they confirmed their hypothesis by mapping cardiac MRI parameters. They found higher global extracellular volume values in the PM group compared to the DM group. The number of affected ventricular segments in PM patients was higher than in DM patients. In our study, 3 of 11 patients had intramyocardial late gadolinium enhancement. Of them, 2 were diagnosed 3 months ago and the other patient was following for 12 months after the diagnosis. All three patients were in clinically inactive stage.

Due to the rare nature of the disease, there is no randomized controlled study on this subject. Accurate and timely treatment may prevent permanent cardiac damage in patients with JDM. Long-term follow-up of patients with JDM have shown frequent cardiovascular complications such as coronary artery disease, hypertension, atherosclerosis in adolescence and adulthood. Despite the fact that JDM can often cause significant cardiovascular morbidity and mortality during the long-term course of the disease, cardiac involvement is often overlooked at the onset of the disease. A cardiac MRI study by ref. ^41^ showed that myocardial LGE on contrast-enhanced magnetic resonance imaging of 4 adult patients with idiopathic inflammatory myopathies had markedly regressed after 6 months of corticosteroid and immunosuppressive therapy. They suggested that patients with cardiac involvement may require more aggressive treatments. IVIG is an anti-inflammatory and immunomodulatory drug commonly used in myocarditis to reduce the damage of inflammatory cytokines in cardiac myositis.^42^ In our study, IVIG was given to 2 patients after cardiac MRI while one could not be treated with IVIG due to financial problems.

Late gadolinium enhancement on cardiac MRI may be seen in various cardiac diseases. Cardiomyopathies, autoimmune and inflammatory diseases, and metabolic and storage diseases cause LGE on cardiac MRI. Accumulation of amyloid fibrils in amyloidosis and glycosphingolipids in Anderson-Fabry disease deteriorate myocardial structure and cause intramyocardial fibrosis. Viral myocarditis, sarcoidosis, and systemic sclerosis are other causes of intramyocardial fibrosis due to inflammation with ischemic damage. Intramyocardial fibrosis and LGE can be found in arrhythmogenic right ventricular cardiomyopathy and dilated or hypertrophic cardiomyopathies.^43^ Myocardial segments involved by contrast media and the pattern of gadolinium distribution may vary from patient to patient even if they have the same disease. Therefore, the clinical value of LGE distribution patterns in providing precise diagnosis remains to be clarified. In our 2 patients, intramyocardial LGE was present in the left ventricular apex. The remaining patient had more diffuse myocardial contrast enhancement (basal anterior, basal anteroseptal, basal anterolateral, basal inferolateral, mid inferior, mid inferoseptal, mid inferolateral, and apical septal segments).

Although conventional echocardiography and ECG findings were completely normal, myocardial involvement was detected in 3 patients in our study. All patients underwent repeat cardiac MRIs. Intramyocardial LGE was resolved in both patients after 6 months of treatment. A similar result was present in Allanore et al.’s study.^41^

The small sample size is one of the important limitations of the study. Furthermore, all patients were at different periods of the disease. Another limitation is that our findings are not enough to make a contribution to predictive factors for cardiac involvement. Cardiac MRIs are difficult to implement in childhood patients. Unfortunately, it was inevitable that motion artifacts would occur. Changes seen may be the result of slow flow within the trabeculae and could be artifacts. However, in the present study, complete myocardial involvement suggests actual myocardial involvement rather than an artifact. Furthermore, there was an absence of involvement in the basal segment of the left ventricular free wall. Thus, the present involvement should not be construed as an artifact. Additionally, myocardial suppression was performed via a look-locker program that provides different TI values, and the best one was chosen. However, due to motion artifacts and high cardiac rate, the images included were a little inferior quality. Therefore, we believe that further studies are needed to decide on which JDM patients should be evaluated by cardiac MRI, and further studies in a larger cohort are needed to confirm our results.

## Conclusion

Juvenile dermatomyositis is a multisystemic disease characterized by diffuse inflammation of skin and muscles. Cardiac involvement may occur in the course of the disease, however, remains unrecognized due to the lack of overt clinical manifestations in childhood. When considering that long-term cardiovascular complications seen in patients with JDM have significant effects on morbidity and mortality, early detection of cardiac involvement by cardiac MRI and aggressive treatment of these patients may improve the long-term prognosis of patients with JDM.

## Supplementary information

Supplementary Figures

## Data Availability

All data generated or analyzed during this study are included in this published article. For additional information concerning these data, the corresponding author can be contacted.
