# Supplementary material for: Cardiac evaluation of patients with juvenile dermatomyositis
Source: Pediatr Res. 2024 Jun 22;97(1):333–40. doi: 10.1038/s41390-024-03336-8 (PMC11798830; doi:10.1038/s41390-024-03336-8)
Supplement: Supplementary file 1 — Supplementary Figures [file 41390_2024_3336_MOESM1_ESM.pdf]

Supplementary figure 1. Left ventricular (LV) segmentation[1].

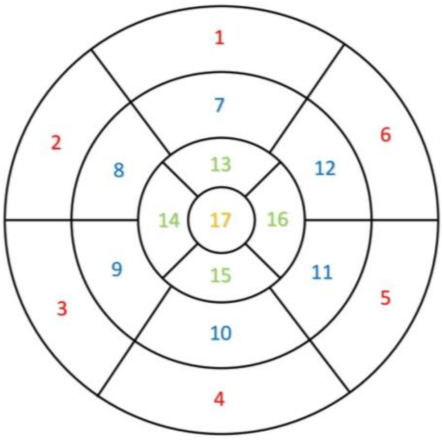

1	Basal anterior	10	Mid inferior
2	Basal anteroseptal	11	Mid inferolateral
3	Basal inferoseptal	12	Mid anterolateral
4	Basal inferior	13	Apical anterior
5	Basal inferolateral	14	Apical septal
6	Basal anterolateral	15	Apical inferior
7	Mid anterior	16	Apical lateral
8	Mid anteroseptal	17	Apex
9	Mid inferoseptal		

Supplementary figure 2. The schematic analyses of the systematic literature review[2].

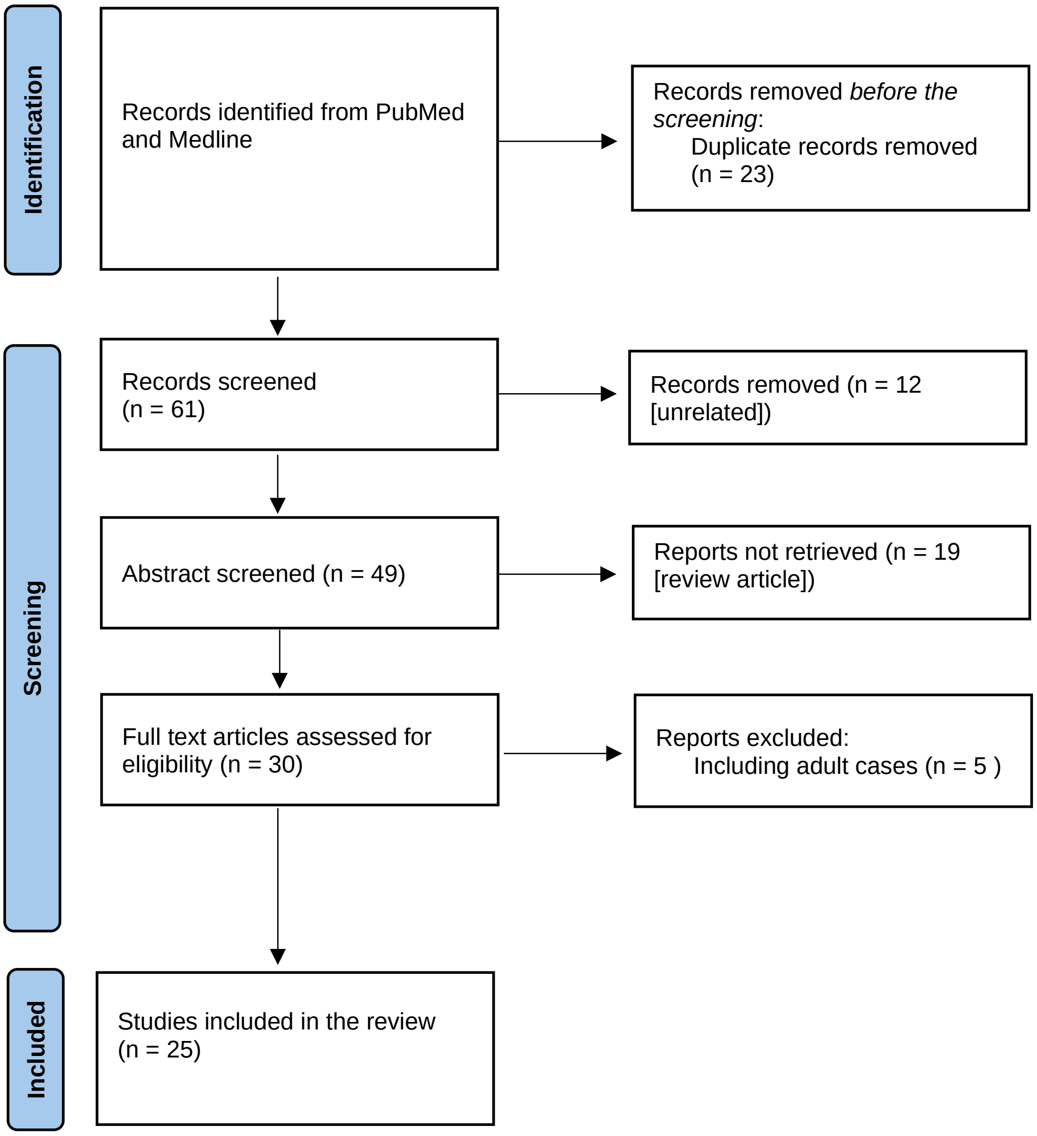

References

1) Cerqueira MD, Weissman NJ, Dilsizian V, Jacobs AK, Kaul S, Laskey WK, Pennell DJ, Rumberger JA, Ryan T, Verani MS; American Heart Association Writing Group on Myocardial Segmentation and Registration for Cardiac Imaging. Standardized myocardial segmentation and nomenclature for tomographic imaging of the heart. A statement for healthcare professionals from the Cardiac Imaging Committee of the Council on Clinical Cardiology of the American Heart Association. Circulation. 2002 Jan 29;105(4):539-42.
2) Page MJ, McKenzie JE, Bossuyt PM, Boutron I, Hoffmann TC, Mulrow CD, et al. The PRISMA 2020 statement: an updated guideline for reporting systematic reviews. BMJ 2021;372:n71.
